# A Rare Case of an Irreducible Patella Dislocation

**DOI:** 10.1155/2016/3728425

**Published:** 2016-10-20

**Authors:** Dimitri E. Delagrammaticas, Scott D. Cordes

**Affiliations:** Department of Orthopaedic Surgery, Northwestern University Feinberg School of Medicine, 676 N. Saint Clair St No. 1350, Chicago, IL 60611, USA

## Abstract

Reports of irreducible patellar dislocations are exceedingly sparse throughout the literature. Obvious radiographic or physical exam findings including fracture or inversion of the patella are often present to explain the block to reduction. Not described previously in the literature is the instance of an irreducible patella dislocation in the setting of innocuous appearing injury imaging. We present a case of a healthy thirty-two-year-old female who sustained an irreducible lateral patella dislocation while participating in a dance aerobics class. Closed means of reduction were unsuccessful, necessitating open reduction. Intraoperative findings suggest incarceration of a nondisplaced fracture and a chondral defect as the block to reduction. Following open reduction, the patient has had no further episodes of pain or instability related to the patella at one-year follow-up. Irreducible patellar dislocations are exceedingly rare injuries, where associated osseous or chondral lesions may necessitate open reduction despite innocuous appearing initial imaging. A high index of suspicion to proceed with open reduction may limit repeated attempts at closed reduction and further injury.

## 1. Introduction

The patella is a large sesamoid bone located in the anterior portion of the knee, tracking within the trochlea of the femur to provide a mechanical advantage to the extensor mechanism of the leg. Acute dislocation can be the result of minor trauma, often occurring after a direct blow or a twisting mechanism during physical activity or sporting events. For the vast majority of acute patella dislocations, spontaneous reduction occurs immediately; however, about 20% of patients may require a reduction for a persistently dislocated patella [1]. Reports of a patella unable to be reduced by closed means are sparse throughout the literature. We present a case of an acute first time patella dislocation in a 32-year-old female, which was unable to be closed reduced necessitating open reduction.

The patient was informed that data concerning the case would be submitted for publication, and she provided consent.

## 2. Case Report

**Figure 1:**
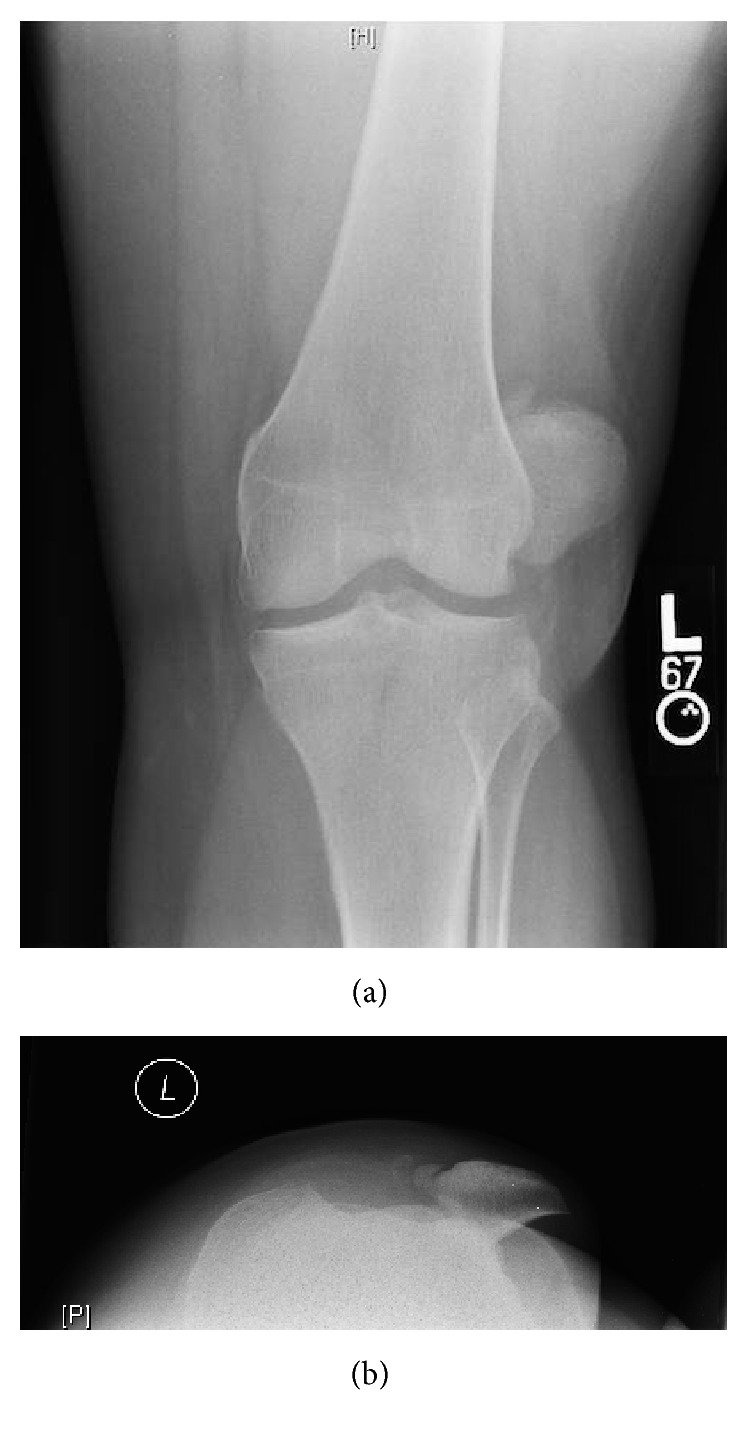
Anterior-posterior and Laurin views of the left knee demonstrating a lateral patella dislocation. Evident in both views is a small fracture of the superior medial aspect of the patella, which does not appear to directly engage the lateral femoral condyle.

An otherwise healthy 32-year-old female presented to the emergency room complaining of severe left knee pain and inability to move her knee through a range of motion after twisting awkwardly on a planted left foot during a dance aerobics class. Upon arrival she was nonambulatory with physical exam findings suggestive of lateral patella dislocation. The patella was palpated laterally and the knee locked at 20 degrees of flexion, with pain at any attempt at motion of the knee. Further exam revealed no other signs of global joint or soft tissue laxity. She did not endorse any signs or symptoms of previous patellar subluxation, instability, or hypermobility. Plain radiographs were obtained in the emergency department confirming the diagnosis of a lateral patellar dislocation with a 9 mm avulsion fracture of anterior medial border of the patella (Figures 1(a) and 1(b)).

**Figure 2:**
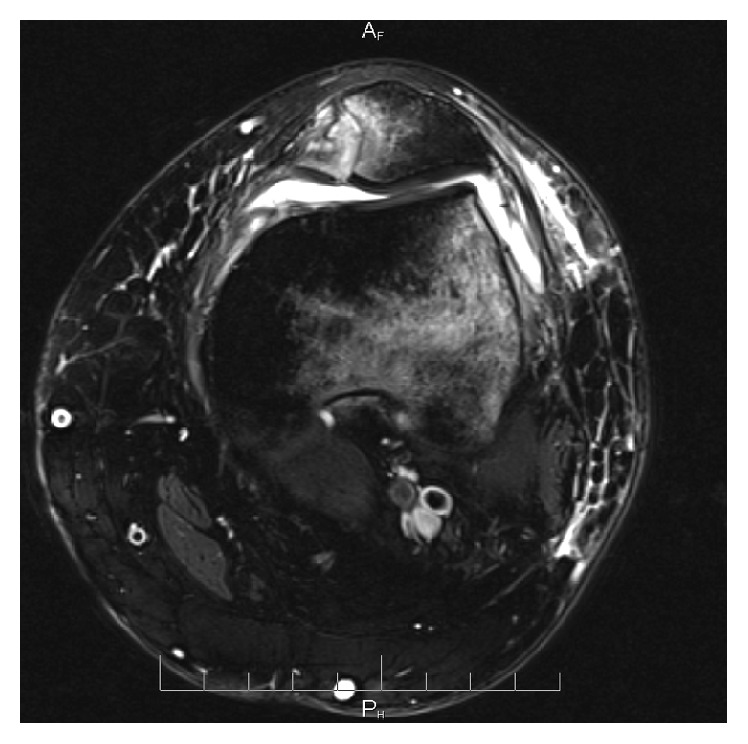
Axial cut of a T2 weighted MRI obtained at follow-up which demonstrates an acute fracture of the medial facet of the patella and characteristic edema of the lateral femoral condyle.

Utilizing intraarticular local anesthetic injection as well as intravenous analgesia and conscious sedation, multiple attempts at closed reduction were made in the emergency room. Maneuvers with flexion, extension, and manual manipulation of the patella were unsuccessful in reducing the patella. This was followed by the use of bone reduction forceps in a percutaneous manner under sterile conditions to attempt to more directly manipulate the patella; however this too was unsuccessful. The patient was subsequently taken to the operative room that day where a closed reduction was attempted, now with complete muscle relaxation under the control of general anesthesia, and again no successful reduction could be obtained in a closed manner. A 2 cm incision along the lateral border of the patella was made where manual exploration revealed that the patella was incarcerated in the lateral patellar retinaculum and locked against the lateral femoral condyle. Reduction of the patella was completed with minimal effort after release of the incarcerated lateral tissues. Next, the incision was closed and a diagnostic arthroscopy was performed demonstrating trauma to the lateral femoral condyle and an 11 mm free floating chondral fragment identified in the medial gutter. Inspection of the articular surface of the patella revealed a corresponding chondral defect within the medial facet, which was debrided back to stable articular cartilage. Aside from this defect, no other articular step off was identified. The patient was placed into a hinged knee brace postoperatively and allowed to be weight bearing as tolerated. At postoperative follow-up, MRI was obtained demonstrating a vertically oriented osteochondral fracture measuring 10 mm in greatest dimension with less than 2 mm chondral step off and partial tearing of the medial patellar retinaculum (Figure 2). Given the vertical orientation and nondisplaced nature of the fracture observed at the time of arthroscopy, the decision was made not to apply any internal fixation to the fracture. Analysis of the patient's relevant osseous anatomy revealed a TT-TG of 12 mm. Postoperatively she progressed through rehabilitation in a stepwise manner and, at 1-year follow-up, did not have any further episodes of pain, instability, or recurrent dislocation.

## 3. Discussion

Patellar dislocations are an orthopaedic condition that can be the result of either direct trauma or low energy twisting mechanisms, where the majority of dislocations occur laterally. The reported risk of patellar dislocation is said to be 6 to 7 per 100,000, with higher incidences in the second decade of life [1, 2]. Stability of the patella is the result of a combination of static and dynamic soft tissue restraints, as well as congruent osseous anatomy. Soft tissue abnormalities, such as generalized ligamentous laxity, torn medial patellofemoral ligament, or a weakened vastus medialis obliquus, may cause or result in instability. Variants in lower extremity anatomy and alignment, including high *Q* angles, genu valgum, or patellofemoral dysplasia, can contribute to patellar maltracking and additionally induce patellar instability. Other osseous abnormalities, such as patella alta or a tibial tubercle-trochlear groove (TT-TG) distance of >20 mm, have also been implicated as factors in acute as well as recurrent dislocation and instability of the patella.

Although patellar instability is a relatively common complaint seen in orthopaedic practice, especially in young active individuals, the vast majority of patellar dislocations spontaneously reduce, with the remainder typically easily reduced in a closed fashion. The case presented here illustrates an instance of an irreducible patella dislocation in a patient sustaining an osteochondral and medial avulsion fracture. There are less than a dozen published reports of laterally dislocated irreducible patella requiring open reduction that can be found. Examples of an irreducible patella as a result of the patella rotating 90 degrees along the vertical axis have been described by several authors [3–6]. Corso presented a case of a 16-year-old boy who sustained a laterally directed blow to his knee, resulting in the patella dislocating and rotating 90 degrees in the vertical plane becoming wedged against the lateral femoral condyle. An open reduction through a medial parapatellar incision was utilized to dislodge the patella anteriorly into an anatomic position. ElMaraghy and Michels each similarly reported vertically rotated irreducible patella dislocations; however, these cases were not the result of a direct blow to the knee but rather a result of low energy indirect mechanisms, similar to the mechanism of our patient presented in this case. Degenerative osteophyte formation along the lateral femoral condyle has been implicated as a block to reduction in older patients, caused by the patella getting locked along the abnormally prominent lateral condyle [7, 8]. Others have reported instances where the irreducibility of a patella dislocation was associated with fracture. Hackl et al. [9] reported a case of a 53-year-old patient who sustained a lateral dislocation with a bony avulsion of the medial structures after a fall from a chair. The resulting medial patellar fracture became impacted into the lateral femoral condyle requiring an open reduction. Yerimah et al. [10] presented a case of a 21-year-old male who required open reduction for an irreducible lateral dislocation secondary to an osteochondral fracture of the patella. Intraoperatively, it was discovered that the rough edge of the osteochondral defect had become locked into the lateral femoral condyle, described as akin to a Hill-Sachs lesion seen in the shoulder. To our knowledge this is the only other case that has been reported of an irreducible patella in a young patient in which a chondral defect became the block to reduction. Unique to this type of injury, in which a chondral defect blocks reduction, is the radiographically innocuous appearing injury imaging. In the case presented here, injury imaging demonstrated only a small avulsion fracture, not engaged on the femoral condyle and likely not accounting for the irreducibility. Postreduction MRI furthermore failed to demonstrate much to attribute the irreducibility of the injury other than a nondisplaced vertically oriented medial facet fracture. We theorize that this fracture became incarcerated within the extensor mechanism and lateral retinaculum and that once the soft tissues were released from the fracture site, the patella was easily reduced into anatomic position.

This case serves to add to the literature of the rare instances where open reduction of an acute patella dislocation is required, in this instance, for what was likely an incarcerated fracture and a chondral defect. While open reduction of this injury was the obvious management given the irreducibility of the patella, what is peculiar about this case and important to consider when treating these injuries is that block to reduction may not be obvious on initial imaging or exam, and a high index of suspicion to proceed with open reduction or early advanced imaging may limit aggressive attempts at closed reduction and further injury. At one-year follow-up, our patient has demonstrated superb outcome, with no residual episodes of instability or pain, symmetric full range of motion, and complete return to activity. Irreducible patellar dislocations are exceedingly rare injuries, where associated osseous or chondral lesions may necessitate open reduction despite innocuous appearing imaging.
